# Working Women in Large Towns

**Published:** 1890-07-12

**Authors:** 


### Working Women in Large Towns.

### VI.—Needlewomen—Fur Sewers, &c.

#### Needlewomen.

This industry is in several respects analogous to the last-mentioned one—tailoring. Each is largely affected by home-work; each contains highly skilled and perfectly unskilled workers; each, consequently, can show us astounding variations in wages. In the work-rooms of Messrs. Stapley and Smith, of which brief mention was made in an earlier article, the best workers are said to earn a pound a week, and—a most important consideration—work is absolutely constant. Collar-making, again, which is done almost entirely in work-rooms, is well paid, Miss Collet's explanation of these two facts being that collars are worn to be seen, and that it is, therefore, important to have the work supervised and done well. She gives the wages of collar-makers as from eleven to seventeen shillings per week, even in the East-end.

Tie-making is by no means so profitable, the wages being lowered by the competition of home-workers, and by the system of sub-contract, which largely prevails in this business. But the makers of shirts and other underclothing are the worst paid of our needlewomen. Some of the skilled workers may receive a fair day's wage, but it is beyond all doubt that the middle-class needlewoman earns, as a rule, less than the ordinary East-end factory-girl; while an inquiry into the wages of the unskilled shirtmaker leads to such distressing disclosures that, as we shall be obliged to take it up later on, we will pass it by for the present.

#### Fur Sewers.

There are many other industries pursued by factory and workshop hands which might well claim to be described here; but as space is limited, we must only consider one more, that of fur-sewing. The fur trade seems to be mostly in the hands of Jews, who buy up large quantities of skins, and either make them up at their own warehouses, or give them out to East-end "chamber-masters." The "cutters" and "nailers" of furs are nearly always men; but the intermediate worker, the fur-sewer, is always a woman. This is not a lucrative trade; in fact, Miss Collet considers that "fur-sewing is the worst paid industry carried on in workshops in the East-end, with absolutely no exception"; and it does not appear that City workers are much better off. Even those who work on good skins—seal and others—only get from 10s. to 15s. per week, while the less skilled hands who work on the commoner skins never earn more than 9s. And these wages, be it remembered, are only earned for six or seven months in the year, there being little or no work done in the winter. One manufacturing firm makes a practice of securing foreign hands, who have just landed, and will take work at any wage, thus making a living out of their helplessness and inexperience. But while it and some other large firms may be doing well, the smaller chamber-masters in this trade seem to be but little better off than their employés.

The poor fur-sewers work all through the hottest part of the year, in close, unhealthy little rooms, of which the Inspector may not even know the existence; and, considering their long hours and miserable pay, alternating with months of enforced idleness, we can hardly be surprised to hear that these poor women do not bear the best character for morality.

#### Class III.—Home Workers.

We now come face to face with the saddest and most perplexing part of our subject—with the class in which is to be found an amount of abject misery which may well make the "whole head sick and the whole heart faint," not only of the worker, but of those who consider and would fain find remedies for her distress. Of course, there are many in this enormous class who do not claim or need our pity. Numbers of married women, from the wife of the labourer or artisan in full employment, to the wife of the London clerk, work for what may really be called pocket money; and a larger class still—the unmarried daughters of men earning comfortable wages—do the same. But the fact that the competition of these women has, as we shall see hereafter, a terrible effect upon the wages paid to their less fortunate sisters, quite destroys the satisfaction with which we might otherwise regard their lot; and it may be laid down as a general rule (subject, of course, to a few exceptions) that wherever a home-worker has nothing but her own work to depend upon, there may heart-rending poverty and misery be found.

It would be impossible to speak in detail of more than a small fraction of the various industries undertaken by home-workers. Even to make out a complete list of them is more than we have space for; but we may state that they comprise among others—mantle-lining, tailoring, fur-pulling, fur-sewing, fish-curing, the making of sacks, shirts, neckties, boxes, brushes, umbrellas, corsets, artificial flowers, bead trimmings, match-boxes, pipes, necklaces, underclothing, boots, and shoes. Some of these industries being pursued to a large extent in workshops, we have found it more convenient to deal with them elsewhere; such are tailoring, fur-sewing, and the making of boots and shoes. Of these, therefore, we need only say that, as a rule, the work done at home is less skilled, less regular, and worse paid, than any work done by indoor hands. As for the poor vest and trouser makers, many of whom are widows, or married women whose husbands are ill or out of work, they will accept work at prices which would be despised by even a "greener," *i.e.*, a perfectly unskilled foreigner. "If we leave the workshop and step into the home," says Miss Potter, "we may watch girls and women straining every nerve, who cannot earn more than twopence, and must frequently content themselves with three-farthings, for an hour's labour."

Pitiful tales, again, are told of home-workers in the boot and shoe trade. Many women are engaged in binding the uppers of carpet slippers—work which, as Mr. Schloss remarks, "no one could get a living out of;" while with regard to the work and wages of home-workers on "sew-round" goods, we cannot do better than quote a most pitiful tale, told by the author of *Toilers in London*. Considerations of space compel us to condense it somewhat; but here are the salient facts, as told by the worker herself—a widow with four children, all of a school age :—

"I machine babies' patent leather shoes, sateen-lined, with front springs and five buttons. They give me fivepence a dozen ; and out of that I have to spend a halfpenny a dozen for cotton and paste, and pay twopence halfpenny a dozen to the fitter who works with me. The two of us working from six to eight can do six dozen—so that she earns eighteen-pence, and I ninepence ; but I often work from five or six in the morning to ten or eleven at night. For another kind of shoe, at a shilling a dozen, I had to pay threepence-halfpenny for cotton, and sevenpence to the fitter. This was cruel work for the money. We could not do more than three dozen a day, if we worked ever so hard." Surely it would pass the wit of the most accomplished romance-writer among us to invent a tale whose heart-rending pitifulness should match some of the facts in this civilised world of ours !

*( To be continued. )*

